# A high throughput method for total alcohol determination in fermentation broths

**DOI:** 10.1186/s12896-019-0525-7

**Published:** 2019-05-22

**Authors:** Peng Zhang, Hao Hai, Dongxu Sun, Weihua Yuan, Weijie Liu, Ruru Ding, Mengting Teng, Lin Ma, Jun Tian, Caifa Chen

**Affiliations:** 1 School of Life Science, Jiangsu Normal University, Xuzhou, 221116 People’s Republic of China; 2 Key Laboratory for Biotechnology on Medicinal Plants of Jiangsu Province, Jiangsu Normal University, Xuzhou, 221116 People’s Republic of China

## Abstract

**Background:**

The potassium dichromate oxidation method used in determination of alcohols in fermentation has two major disadvantages. This method cannot be used to determine alcohols in raw fermentation broth samples, which often contain various reducing sugars. The method is not environment friendly due to the carcinogenicity of Cr (VI) used.

**Results:**

A new method for determination of reducing sugars and total alcohols in raw fermentation broths was developed. The fermentation broth was pretreated to remove proteins, polysaccharides, glycerol and organic acids. The colorimetric change from both total alcohols and reducing sugars by potassium permanganate oxidation was measured. The portion of colorimetric change from oxidation of reducing sugars was determined by DNS test and subtracted. The remaining portion of colorimetric change was then used to calculate the total alcohol concentration in the sample.

**Conclusions:**

Using this method, total alcohol concentration can be easily and accurately determined in both distilled samples and raw fermentation broth samples. It is fast and environmental friendly.

## Background

Total alcohols and reducing sugar concentrations are two important parameters in fermentation of wine, beer and fuel ethanol [1–3]. It provides information on optimization and regulation of the fermentation process to increase the yield and quality of the products.

Several methods have been used in determination of ethanol concentration, including gas chromatographic methods [4–6], gas chromatography-mass spectrometry [7], gas chromatography combustion isotope ratio mass spectrometry [8], and liquid chromatograph-mass spectrometry [9, 10]. The gas or liquid chromatographic methods require expensive instruments, and are time-consuming, so they cannot be widely used to closely monitor the fermentation process.

Gravimetric methods have also been used in ethanol determination (for example, the method described in the Chinese Standard GB/T 5009.48–2003). In these methods, distillation is a critical step. But as it often takes about 1 h to distill a sample of 100 ml, it is difficult to use these methods in high throughput tests. Ethanol concentration can also be determined using ethanol oxidase or ethanol dehydrogenase, but the results are easily disturbed by the presence of various enzymes in the fermentation broth.

Chemical methods of ethanol determination are based on colorimetric changes upon reactions of chemicals such as potassium dichromate with ethanol [11, 12]. However, two problems are associated with these methods. First, the use of dichromate has been avoided by most of the world because of the carcinogenicity of Cr (VI). Second, the methods cannot be used to determine ethanol concentration in raw fermentation broths due to the presence of various reducing sugars and side-products, which can also react with potassium dichromate.

In the present study, we developed a new method for total alcohols and reducing sugar determination. The principle and the main procedure of this method are as the following. The fermentation broth is pretreated with Ca(OH)_2_ to remove organic acids and glycerol [13]. Organic acids and glycerol can react and precipitate with Ca(OH)_2_. Then, the fermentation broth is treated with trichloroacetic acid (TCA) to precipitate proteins and cell debris in the sample under acidic conditions [14–16]. The precipitated materials are removed by a simple centrifugation step. The supernatant is then treated with hexadecyltrimethylammoniumbromide (CTAB) to precipitate polysaccharides and the remaining residual proteins [17], which are removed by centrifugation. After these steps, the main interfering substance still present in the sample are reducing sugars, which are oxidized by addition of 3, 5-dinitrosalicylic acid (DNS) under alkaline conditions with heating [18], and quantitatively measured by colorimetric detection. Meanwhile, the sample is reacted with potassium permanganate (we used potassium permanganate as a replacement of potassium dichromate to avoid its carcinogenicity) in a parallel test, in which the color change from both total alcohols and reducing sugars in the sample is quantitatively measured. After subtracting the portion of absorbance increase contributed by reducing sugars, the remaining absorbance decrease can be used to calculate the total alcohol concentration in the sample.

Using this method, total alcohol concentration can be easily determined in both distilled samples and raw fermentation broth samples in a high throughput manner.

## Results

### Linearity and detection limits

#### The linearity and detection limit of glucose in reaction with DNS

**Fig. 1:**
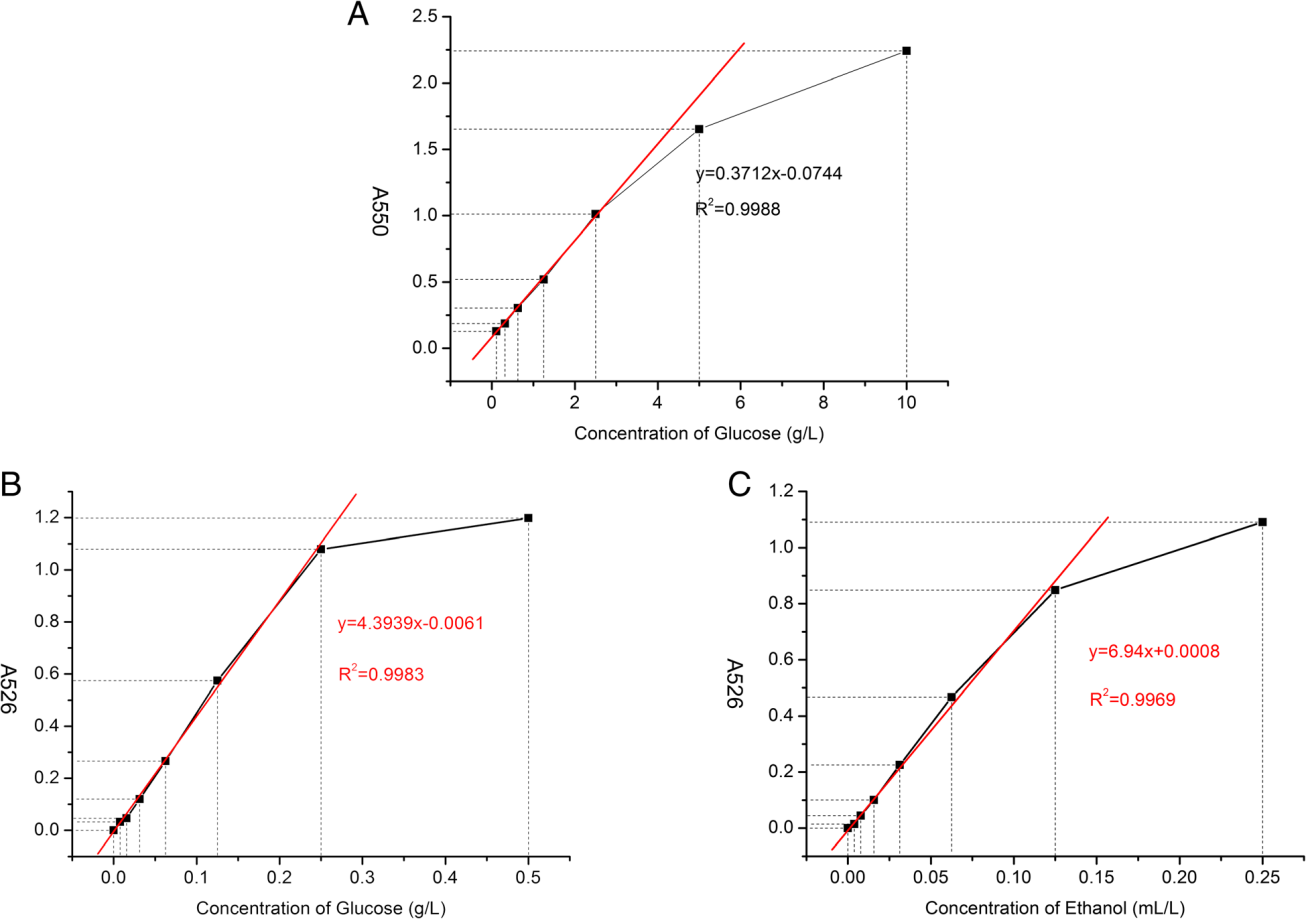
Standard curves of glucose and ethanol (**a** DNS method for glucose; **b** potassium permanganate method for glucose; **c**: potassium permanganate method for ethanol; A526 = A526 before the reaction – A526 after the reaction)

Various concentrations of glucose solutions (2-fold serial dilutions from 10 g L-1 to 0.15625 g L-1) were prepared to determine the standard curve in reaction with DNS and A550 was determined. The glucose standard curve thus generated. It showed a linear range from 2.5 g L-1 to 0.15625 g L-1, with a regression equation of y = 0.3721x + 0.0744 and R^2^ = 0.9988. When the concentration of glucose exceeded 2.5 g L-1, the A550 increase deviated from the linear standard curve (Fig. 1A).

#### The linearity and detection limit of glucose by method of potassium permanganate oxidization

Various concentrations of glucose (2-fold serial dilutions from 2 g L-1 to 1.96 mg L-1) were prepared to estabolish the standard curve of A526 increase by the potassium permanganate treatment. The standard curve displayed a linear range equal and below 0.25 g L-1, with the regression equation y = 4.3939x-0.0061, and R^2^ = 0.9983. When the concentration of glucose was above 0.25 g L-1, the A526 increase deviated from the linear standard curve (Fig. 1B).

#### The linearity and detection limit of ethanol by method of potassium permanganate oxidization

Various concentrations of ethanol (2-fold serial dilutions from 2 mL L-1 to 1.96 μL L-1) were prepared to estabolish the standard curve of A526 increase by the potassium permanganate treatment. A linear range was observed at ethanol concentrations equal and below 125 μL L-1, with y = 6.94x + 0.0008 and R^2^ = 0.9969. However, when the concentration of ethanol exceeds 125 μL L-1, The A526 increase deviated from the linear standard curve (Fig. 1C).

### The progression of reactions and product stability

For broad application of our new method in ethanol determination, it is important that the chemical reactions proceed to the completion and the concentrations are in the linear range when the reaction is stopped, and that the products are stable. We therefore tested the product stability in our new method.

**Fig. 2:**
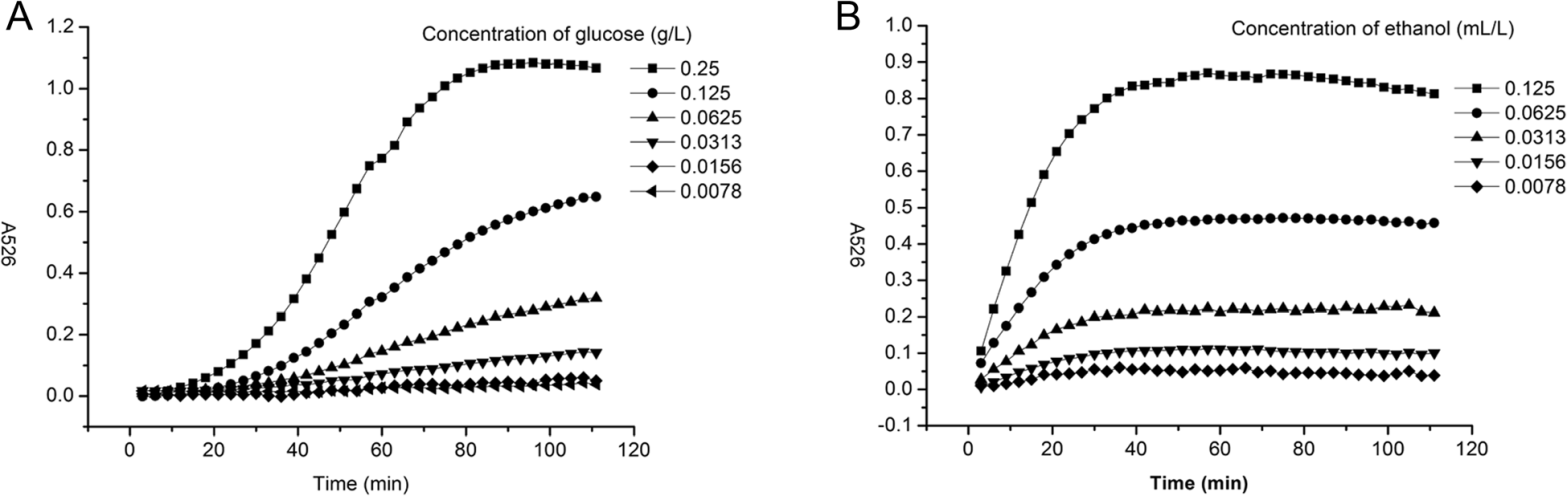
The stability of oxidization (**a** The stability of DNS oxidization; **b** The stability of potassium permanganate oxidization; A526 = A526 before the reaction – A526 after the reaction)

One hundred μL of 2-fold serial dilutions of glucose (from 0.25 g L-1 to 0.0078 g L-1) was mixed with 100 μL of potassium permanganate, incubated at 40 °C, and A526 was determined every 3 min. As shown in Fig. 2A, the A526 increase plateaued at 80–100 min when glucose was at 0.25 g L-1. Below 0.25 g L-1, the A526 continued to increase beyond 120 min in a near linear manner.

A similar study on the reaction of ethanol with potassium permanganate indicated that the A526 reached to maximum at 30 min, and stably maintained this level until 120 min. (Fig. 2B).

### The interference of the results

#### Effect of various compounds on glucose determination by the DNS method

**Fig. 3:**
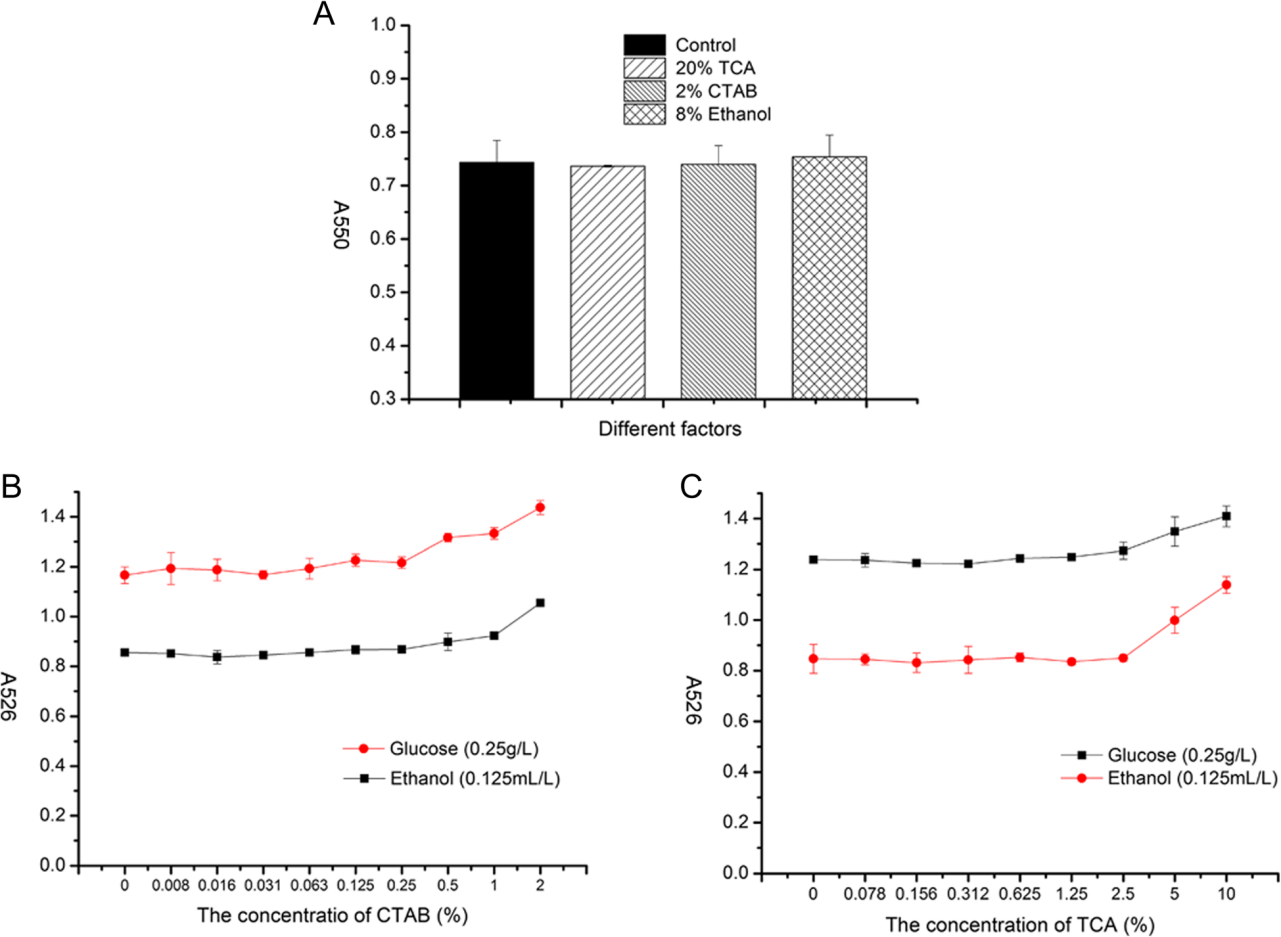
The effect of different factors on the determination of glucose and ethanol (**a** The effect of different factors on the determination of glucose with DNS reaction; **b** The effect of CTAB in potassium permanganate reaction; **c** he effect of trichloroacetic acid in potassium permanganate reaction; A526 = A526 before the reaction – A526 after the reaction)

As TCA and CTAB were used in the pretreatment of fermentation broths and ethanol was produced, the effect of these compounds on glucose determination by the DNS method was measured. Four tubes of glucose solution at 4 mg mL-1 were mixed with 2%TCA, 2%CTAB, 8% ethanol and distilled water (control) respectively, and proceeded with the DNS method. The results shown in Fig. 3A indicate that there was no significant difference between the control and the samples containing the compounds tested by statistical analysis.

#### The effect of CTAB on ethanol determination by potassium permanganate

Different concentrations of CTAB were added into 0.125 mL L-1 ethanol or 0.25 g L-1 glucose. These samples were treated as2.4. The variation of A526 was showed in Fig. 3B. CTAB cannot disturb the result when the concentration of CTAB is under 0.25%.

#### The effect of TCA on ethanol determination by potassium permanganate

A similar test on the effect of various concentrations of TCA on ethanol determination by potassium permanganate treatment was performed. The A526 increased significantly, when the concentration of TCA exceeded 2.5% (Fig. 3C). In this method, we pretreated our samples with 10% TCA, but when we determinate it after 10 to 100 times dilute. Therefore, the TCA concentration is below 1%. So TCA and CTAB can be used in this method.

### Testing the accuracy of the new method

The accuracy of detection is very important for a new method. We spiked ethanol to a pretreated sample at various concentrations and calculated the increase in the ethanol concentration as determined by the new method. The results are showed in Table 1. All the 10 concentrations of ethanol spiked showed high detection accuracy, with the difference between the spiked amount and the calculated increase by our new method being less than 10%. Out of the 10 spiked samples, 8 showed the differences of less than 2%. These results indicate that the new method is very accurate.

**Table 1:** Determination of accuracy of the RSSAA method

NO.	Ethanol concentration spiked	Ethanol concentration detected^a^	Detection accuracy
	(%,V/V)	(%,V/V)	(%)
1	1.93	2.01 ± 0.09	104.1 ± 4.7
2	3.79	3.85 ± 0.12	101.6 ± 3.2
3	5.57	5.98 ± 0.28	107.4 ± 5
4	7.3	7.32 ± 0.15	100.3 ± 2.1
5	8.95	9.08 ± 0.09	101.5 ± 1
6	10.55	10.61 ± 0.11	100.6 ± 1
7	12.11	12 ± 0.12	99.1 ± 1
8	13.59	13.72 ± 0.25	101 ± 1.8
9	15.02	15.12 ± 0.21	100.7 ± 1.4
10	16.42	16.44 ± 0.02	100.1 ± 0.1

^a^The values = measured values - 1.5% (ethanol concentration in PS sample)

### Example for our new method

Here we provide an example to demonstrate how to use the new method to determine ethanol concentration. Pretreated sample was prepared from an ethanol fermentation culture as described in the Materials and Methods. A small aliquot of the pretreated sample was diluted 100-fold with water, and 0.1 mL of the diluted pretreated sample was mixed with 0.1 ml of potassium permanganate solution in a 96-well plate. At the same time, the standard curves of ethanol and glucose treated with potassium permanganate were established in the same 96-well plate. The plate was kept at 40 °C for 90 min and A526 was measured. The A526 of the diluted pretreated sample was 0.685. Another small aliquot of the same pretreated sample was diluted 10 fold with water, and 200 μL of the diluent was mixed with 600 μL of DNS and kept at 100 °C for 10 min. After cooling down to room temperature, 200 μL of the mixture was transferred to a 96-well plate and A550 was measured to be 0.2962. Meanwhile, a standard curve of glucose was established with DNS under the same conditions. Based on the regression equation of y = 0.3712x-0.0744 from the glucose-DNS treatment standard curve, the concentration of reducing sugars in the diluted pretreated sample was calculated to be 0.9984 g L-1. Because the pretreated sample was diluted 10-fold, the reducing sugar concentration in the undiluted pretreated sample was 9.984 g L-1. Then, we calculated how much A526 could be generated from 99.84 mg L-1of glucose that was present in the 100-fold dilution of the pretreated sample, by reacting with potassium permanganate. Based on the A526-glucose standard curve treated with potassium permanganate, 99.84 mg L-1 of glucose would generate A526 of 0.4326. By subtracting 0.4326 from 0.6850, the A526 from total alcohols in the pretreated sample was calculated be 0.2524. This A526 value was used to calculate the concentration of ethanol in the pretreated sample using the regression equation of ethanol standard curve, y = 6.94x + 0.0008. The concentration of ethanol in the undiluted pretreated sample was 3.625 ml L-1 after 100-fold conversion.

### Application of the new method to monitor ethanol concentrations during the fermentation process

**Fig. 4:**
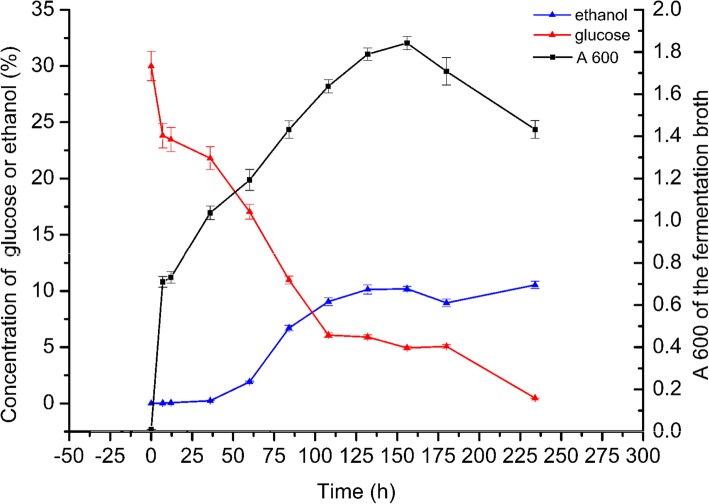
The application of our new method to monitor the concentrations of total alcohols and remaining reducing sugars during a fermentation process

We used the new method to monitor the increase in ethanol yield during the fermentation process by *Z. mobilis* in liquid medium ZM4-G30. Samples were taken daily from the culture. The concentrations of ethanol and remaining glucose were determined with the new method. The results was showed in Fig. 4. When the A600 of *Z. Mobilis* was increased as time went by until 160 h. The concentration of ethanol reached the maximum level. The concentration of glucose went down until the last time we determined.

### Comparison between the new method and other methods

**Fig. 5:**
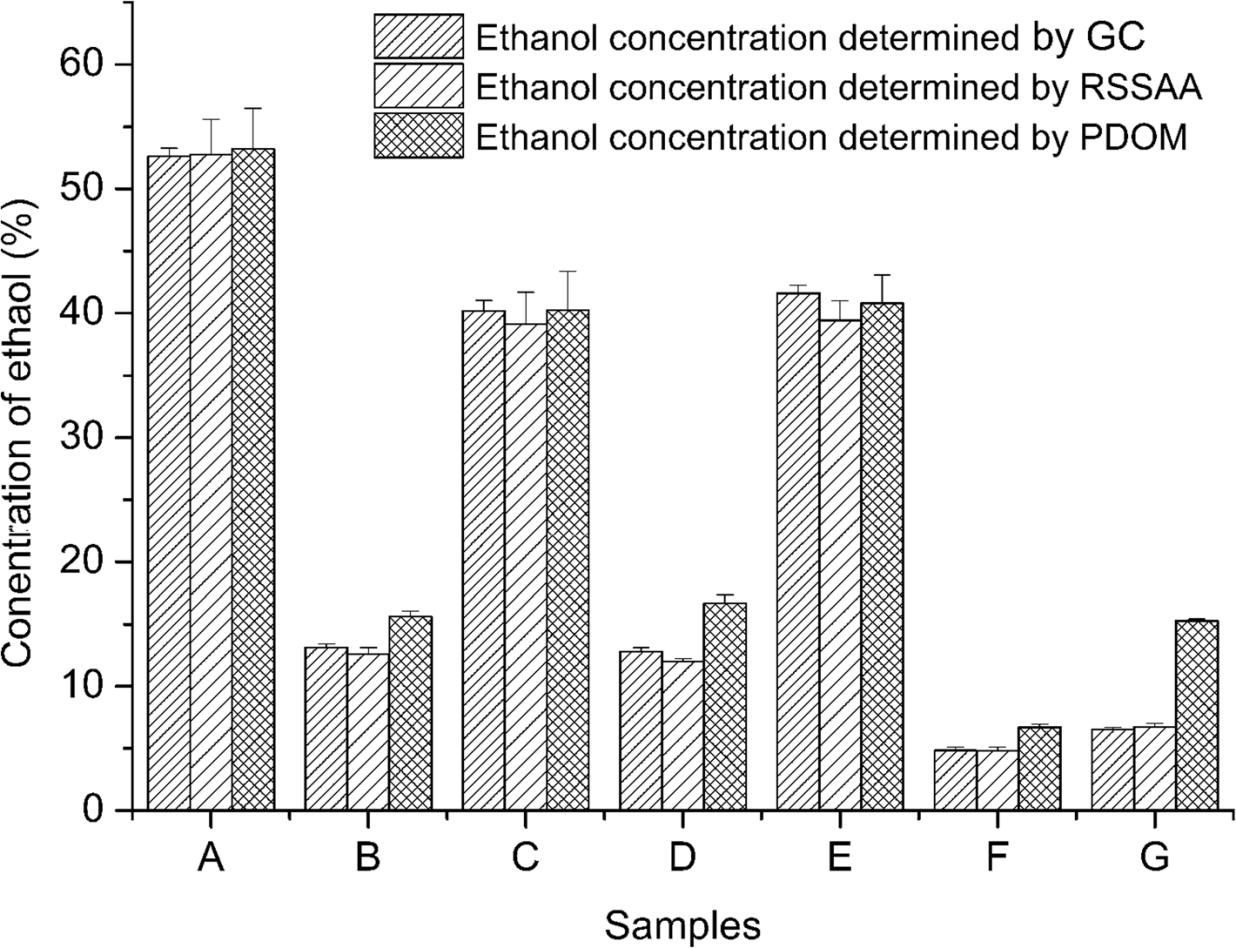
Comparison of the new method and other methods. Maotai wine from China, **b** Jacob’s Creek from Australia, **c** Absolut Vodka from Sweden, **d** Taster red wine from Chile, **e** Macaulay Whisky from Scotland, **f** Qingdao beer from China, **g** fermentation broth)

The new method can be used in determination of ethanol not only in fermentation broths, but also in ethanol containing beverages including distilled spirit, beers and wines. We chose six commercial ethanol-containing beverages and a fermentation broth to determined their ethanol concentration using the new method and other methods (Fig. 5). Ethanol concentration was also analyzed by gas chromatography (Shandong Ruihong, SP-6890, China), carrier gas: nitrogen, capillary column (SE-54, Agilent, USA), injection temperature 150 °C, flame ionization detector temperature 160 °C; Zheda Zhida Data Processor) and acetone was used as an internal standard (modified from [19]). The ethanol concentrations of three brands of distilled spirit were all in agreement with the ethanol concentrations analyzed by GC. However, they are quite different between potassium dichromate oxidation method and our new method in beers, wines and fermentation broth. This result indicates that the new method can be used in the ethanol concentration determination of ethanol containing beverage and fermentation broth. The new method is more precise and reliable than potassium dichromate oxidation method.

## Discussion

The new method of determining alcohol and reducing sugars has several advantages over other methods currently used in the industry. First, it requires less volume of sample for detection. Using the new method, pretreated fermentation broth samples are usually diluted 10–20 folds, therefore only 100 μl raw fermentation broth samples were needed. On the other hand, in gravimetric methods (GB/T 5009.48–2003), the volumes of sample to be distilled are usually at least 100 ml. If the ethanol concentration in the raw fermentation broth is 10%, it requires about 1000 ml of fermentation broth. If the concentration of ethanol is under 1%, more than 10 L fermentation broth is required. Second, our new method is more accuracy and environment friendly than the potassium dichromate oxidation method. The potassium dichromate oxidation method [11, 12] is based on colorimetric changes upon chemical reactions with ethanol. This method cannot be directly used in determination of ethanol concentration in raw fermentation broths because of the presence of reducing sugars that can also react with potassium dichromate. With our new method, the noise caused by the reducing sugars in fermentation broths is effectively measured and subtracted from the total signal, allowing accurate determination of ethanol in the sample. However, potassium permanganate can react with non-reducing sugar, which cannot be detected by DNS. Therefore, this method can only be applied in the ethanol fermentation in which reducing sugars or polysaccharides of reducing sugars was used as carbon source. Our new method is more accurate than potassium dichromate method because the results of this method is not interfered by the presence of TCA, CTAB and other materials. In the new method, we used potassium permanganate as a replacement of potassium dichromate in the chemical reaction with ethanol and reducing sugars, thus avoiding the problem of carcinogenicity of Cr (VI). Therefore, the new method is more environment friendly.

Third, the new method is simply, fast, of low cost, and can be used in a high throughput manner. In this aspect, the new method has clear advantages over various gas- and liquid-chromatographic methods, which, although pretty accurate, are usually complicated, slow, of high cost, and cannot be used in high throughput tests.

Fourth, in monitoring the process of ethanol fermentation, the concentrations of ethanol and reducing sugars are two important parameters. With the new method, not only the concentration of ethanol, but also that of reducing sugars, can be simultaneously determined. These results provide information in optimizing and regulating the fermentation processes.

There are some disadvantages in our new method. Potassium permanganate is not stable. It can react with water at low pH and complicate the test results. To minimize this disadvantage, the potassium permanganate solution should be prepared right before it is used and kept in dark. The standard curves of ethanol and glucose from the potassium permanganate treatment should be simultaneously established with the experiment samples.

## Conclusions

A new analytic method was established to determine total alcohol concentration in fermentation broths. There are many advantages in this method: No precision testing instrument is needed. The results are reliable and precise. The operation process is easy and simple. Less volume of samples and reagents is required. The method is more environmental friendly. Multiple samples can be easily processed at a time.

## Methods

### Medium and reagents

ZM4 medium was prepared by dissolving 20 g of glucose, 10 g of yeast extract and 2 g of monobasic potassium phosphate in water to 1000 ml and the pH was adjusted to 6.0. ZM4-G30 fermentation medium was made by adding glucose into the ZM4 medium to a final concentration of 30%. All the media were sterilized by autoclaving at 121 °C for 30 min.

DNS solution contains 6.3 g DNS, 262 mL NaOH solution (2 M), 185 g Potassium sodium tartrate, 5 g crystallization of phenol, 5 g sodium sulfite in 1000 mL. DNS solution should be kept in dark for a week at least before use.

Potassium permanganate solution contains 0.395 g potassium permanganate, 10 g Sodium tetraborate and 250 mL sulfuric acid (98%).

Absolute ethanol, TCA, CTAB and glucose were all purchased from Sinopharm in China.

### Strain

*Zymomonas mobilis* strain ZM4 (ATCC31821), which has been widely used in ethanol fermentation studies, was purchased from American Type Culture Center (ATCC).

### Sample preparation and pretreatment

Ethanol fermentation by *Z. mobilis* was carried out in ZM4-G30 fermentation medium [3]. The fermentation cultures were centrifuged at 13,800 g for 5 min at 4 °C. The supernatants were mixed with isovolumetric 20% TCA and kept at room temperature for 5 min. Then the mixtures were centrifuged at 13,800 g for 5 min. The supernatant was filtered with a bacterial filter (φ0.22 μm). The supernatants were mixed with 1/5 volume of 20% CTAB, kept at 65 °C for 10 min, then centrifuged at 13,800 g for 10 min. The supernatants were thereafter called pretreated samples. Two aliquots of the pretreated sample were taken, one for ethanol determination and another for reducing sugar determination.

### DNS treatment

One pretreated sample aliquot was treated with DNS under alkaline conditions to determine the concentration of reducing sugars. The pretreated sample was diluted 10-fold with water. The diluent (200 μL) was mixed with DNS (600 μL) and kept at 100 °C for 10 min. After cooling down to room temperature, 200 μL of the mixture was transferred to a 96-well plate. Absorbance at 550 nm was determined.

### Potassium permanganate treatment

Another pretreated sample aliquot was diluted 100-fold with water and 100 μL of the diluted samples was added to a 96-well plate. Then 100 μL of potassium permanganate solution was added, mixed and kept at 40 °C for 90 min. Absorbance at 526 nm was determined.

### Test of completion of the reaction

To determine the completion of the colorimetric reactions and the stability of the product, glucose and ethanol at various concentrations were treated with potassium permanganate solution as described in 2.4 and A526 was determined every 3 min up to 120 min.

### Result calculation

The concentration of ethanol in fermentation broth was calculated in 5 steps. Step 1, the A526 generated from the addition of potassium permanganate in the pretreated sample was determined as described in method 2.4 above, and recorded as ‘A’. Step 2, the concentration of reducing sugars in the pretreated sample was calculated using the A550 generated in the DNS method as described in method 2.3 above, based on the glucose-DNS standard curve. Step 3, the portion of A526 contributed by the reducing sugar in the sample was calculated according to the standard curve of potassium permanganate method for glucose and recorded as ‘B’. Step 4, the portion of A526 contributed by ethanol in the sample was calculated by subtracting B from A. Step 5, the concentration of ethanol in the sample was calculated based on the standard curve of potassium permanganate reaction for ethanol.

## Abbreviations

CTAB: hexadecyltrimethylammoniumbromide
DNS: 3, 5-dinitrosalicylic acid
TCA: trichloroacetic acid
